# Predicting cancer type from tumour DNA signatures

**DOI:** 10.1186/s13073-017-0493-2

**Published:** 2017-11-28

**Authors:** Kee Pang Soh, Ewa Szczurek, Thomas Sakoparnig, Niko Beerenwinkel

**Affiliations:** 1 Department of Biosystems Science and Engineering, ETH Zurich, Mattenstrasse 26, Basel, 4058 Switzerland; 2 Saw Swee Hock School of Public Health, National University of Singapore, Tahir Foundation Building, 12 Science Drive 2 MD1, Singapore, 117549 Singapore; 3 Institute of Informatics, University of Warsaw, Banacha 2, Warsaw, 02-097 Poland; 4 Biozentrum, University of Basel, Klingelbergstrasse 50/70, Basel, 4056 Switzerland; 5 SIB Swiss Institute of Bioinformatics, Basel, 4058 Switzerland

**Keywords:** Cancer genomics, Cancer-type prediction, Cancer diagnostics, Machine learning, Personalised medicine

## Abstract

**Background:**

Establishing the cancer type and site of origin is important in determining the most appropriate course of treatment for cancer patients. Patients with cancer of unknown primary, where the site of origin cannot be established from an examination of the metastatic cancer cells, typically have poor survival. Here, we evaluate the potential and limitations of utilising gene alteration data from tumour DNA to identify cancer types.

**Methods:**

Using sequenced tumour DNA downloaded via the cBioPortal for Cancer Genomics, we collected the presence or absence of calls for gene alterations for 6640 tumour samples spanning 28 cancer types, as predictive features. We employed three machine-learning techniques, namely linear support vector machines with recursive feature selection, *L*_1_-regularised logistic regression and random forest, to select a small subset of gene alterations that are most informative for cancer-type prediction. We then evaluated the predictive performance of the models in a comparative manner.

**Results:**

We found the linear support vector machine to be the most predictive model of cancer type from gene alterations. Using only 100 somatic point-mutated genes for prediction, we achieved an overall accuracy of 49.4±0.4 *%* (95 % confidence interval). We observed a marked increase in the accuracy when copy number alterations are included as predictors. With a combination of somatic point mutations and copy number alterations, a mere 50 genes are enough to yield an overall accuracy of 77.7±0.3 *%*.

**Conclusions:**

A general cancer diagnostic tool that utilises either only somatic point mutations or only copy number alterations is not sufficient for distinguishing a broad range of cancer types. The combination of both gene alteration types can dramatically improve the performance.

**Electronic supplementary material:**

The online version of this article (doi:10.1186/s13073-017-0493-2) contains supplementary material, which is available to authorized users.

## Background

Histopathologic examination of tumour tissues remains the main method for diagnosing cancer in most cases. When preliminary signs and symptoms indicate the possibility of tumour growth in a patient, a biopsy is carried out to extract tissue samples from the patient for visual examination under a microscope by a pathologist. If the cells are identified as cancerous, they are categorised into known cancer subtypes, based on the site where the cancer originates. Establishing the cancer type and site of origin is important, because it helps to determine the most appropriate course of treatment for cancer patients. Unfortunately, there are patients with cancer of unknown primary, where the site of origin of the cancer cannot be established from an examination of the metastatic cancer cells. Most patients with cancer of unknown primary have poor survival [1]. Since cancer is a genetic disease [2] and progresses from normal cells via acquisition of somatic alterations [3], a genomic analysis of tumour DNA could potentially be used to identify cancer types and primary sites.

There have been numerous studies on cancer-type identification through genomic analysis. Early work in this area relied on gene expression profiles of patients to differentiate between a relatively small number of cancer types or subtypes [4–7]. More recent work used DNA methylation profiles to predict the tissue of origin [8, 9]. Other studies on the broader cancer genomic landscape are also revealing. For example, Ciriello et al. identified a tree-like stratification of cancer types and their oncogenic signatures, based on somatic mutation, copy number alterations and methylation in tumour DNA [10]. From a joint analysis of cancer data sets and data generated by the Epigenome Roadmap consortium, Polak et al. found a relationship between the chromatin organisation of primary cancer cells and the distribution of mutations along their cancer genome [11]. These studies uncovered the presence of genomic differences among cancer types, thus pointing to the feasibility of predicting the primary tumour tissue of origin from genomic data. In this paper, we evaluate the potential and the limitations of determining the cancer type from a small set of genetic alterations in tumour DNA. Our aim is to develop a genetic alteration-based diagnostic tool for patients who have been diagnosed with cancer, such as when metastases are found, but from whom the primary site remains unknown.

In our computational proof-of-concept study, we make use of sequenced tumour DNA from a cancer genome database to explore subsets of all genes as predictors. The establishment of cancer databases in recent years has afforded an unprecedented opportunity to examine thousands of tumour samples, spanning a wide range of different cancer types, at once. Such large volumes of data allow us to explore the feasibility of identifying the cancer types of tumour DNA based on alterations in the genes, which is a classification problem. We are, however, not just interested in whether tumour DNA can be correctly classified into the respective cancer types. From a cost-effectiveness point of view, a practical cancer diagnostic tool should ideally use as few genes as possible, so that it remains affordable. Hence, we are also interested in whether a high level of accuracy can be achieved using a small number of gene markers. This can be cast as a feature selection problem in machine learning: we will identify and select a small subset of the gene alterations that are most informative about cancer type.

The type of gene alterations we employ to distinguish one cancer type from another are somatic point mutations and copy number alterations. Although studies on cancer types such as colorectal and breast cancer have shown that genetic alterations in tumours are mostly in the form of somatic point mutations [3] and that somatic mutations alone are sufficient for accurately predicting cancer subtypes [12], there are cancer types where mutations seem to occur predominantly in the form of copy number alterations [10]. Hence, we are interested in investigating whether the inclusion of information on copy number alterations can improve our classification results. This interest is also motivated by a reported improvement in the detection of copy number alterations in tumour DNA [13].

Our analysis indicates that linear support vector machines (SVMs), coupled with recursive feature elimination, perform best in a cancer classification task. Moreover, we found that it is crucial to use copy number alterations in addition to somatic point mutations for improved diagnostic performance. With this optimised approach, we found that a gene panel of as few as 50 genes allows cancer-type prediction with an accuracy of around 77 %.

## Methods

### Data collection and preprocessing

The analysed data, consisting of somatic mutations and copy number alterations in 6640 tumour samples from 28 cancer types (Table 1), were downloaded via the cBioPortal for Cancer Genomics [14–16]. Most of the data were generated by The Cancer Genome Atlas (TCGA) Research Network.

**Table 1:** Cancer types and their respective sample sizes

Cancer type (data set)	Class label	Sample size
Bladder urothelial carcinoma (TCGA, Nature 2014) [47]	1	127
Breast invasive carcinoma (TCGA, Cell 2015) [48]	2	973
Colorectal adenocarcinoma (TCGA, Nature 2012) [49]	3	212
Glioblastoma (TCGA, Cell 2013) [50]	4	280
Head and neck squamous cell carcinoma (TCGA, Nature 2015) [51]	5	279
Kidney renal clear cell carcinoma (TCGA, Nature 2013) [52]	6	418
Acute myeloid leukaemia (TCGA, NEJM 2013) [53]	7	190
Lung adenocarcinoma (TCGA, Nature 2014) [54]	8	230
Lung squamous cell carcinoma (TCGA, Nature 2012) [55]	9	178
Ovarian serous cystadenocarcinoma (TCGA, Nature 2011) [56]	10	316
Uterine corpus endometrial carcinoma (TCGA, Nature 2013) [57]	11	240
Adenoid cystic carcinoma (MSKCC, Nat Genet 2013) [58]	12	55
Brain lower grade glioma (TCGA, Provisional)	13	279
Cervical squamous cell carcinoma and endocervical adenocarcinoma (TCGA, Provisional)	14	191
Kidney renal papillary cell carcinoma (TCGA, Provisional)	15	161
Liver hepatocellular carcinoma (AMC, Hepatology 2014) [59]	16	231
Pancreatic adenocarcinoma (TCGA, Provisional)	17	145
Prostate adenocarcinoma (TCGA, Cell 2015) [60]	18	332
Skin cutaneous melanoma (TCGA, Provisional)	19	278
Stomach adenocarcinoma (TCGA, Nature 2014) [61]	20	287
Papillary thyroid carcinoma (TCGA, Cell 2014) [62]	21	399
Adrenocortical carcinoma (TCGA, Provisional)	22	88
Kidney chromophobe (TCGA, Cancer Cell 2014) [63]	23	65
Pheochromocytoma and paraganglioma (TCGA, Provisional)	24	161
Sarcoma (TCGA, Provisional)	25	240
Testicular germ cell cancer (TCGA, Provisional)	26	149
Uterine carcinosarcoma (TCGA, Provisional)	27	56
Uveal melanoma (TCGA, Provisional)	28	80

The data were downloaded via the cBioPortal for Cancer Genomics

We first identified 28 cancer types for our study based on the availability of both somatic mutation and copy number alteration information on the cBioPortal website. We then compiled two separate lists of genes for these cancer types from the same website: somatic point-mutated genes and copy number altered genes. These genes are either recurrently mutated or altered genes detected by the MutSig and GISTIC algorithms [17, 18], or known cancer genes listed in the Sanger Cancer Gene Census [19]. In addition, we added a set of 572 genes from the 76th release of the Catalogue of Somatic Mutations in Cancer (COSMIC) [20] to our list of somatic point-mutated genes.

Next, we compared these two gene lists against a list of pseudogenes and non-coding genes downloaded from the HUGO Gene Nomenclature Committee [21]. We found 20 pseudogenes and non-coding genes among the somatic point-mutated genes and 1054 such genes among the copy number altered genes. Since amplification and deletion of chromosome regions affect the copy number of many genes, which may not all be causally related to cancer, it is not surprising that the GISTIC algorithm picked up many more pseudogenes and non-coding genes among the copy number altered genes. We chose to remove all the pseudogenes and non-coding genes from our gene lists, as we want the genes of our diagnostic gene panel to be interpretable and relatable to other studies.

After trimming the two gene lists, we extracted the gene alteration information for the 6653 tumour samples belonging to the 28 cancer types. The data were arranged into two binary matrices: a somatic point mutation matrix and a copy number alteration matrix. In the former, each column of the matrix corresponds to a somatic point-mutated gene in our gene list, while each row corresponds to a tumour sample. A 1 at position (*i*,*j*) of the matrix indicates that the *j*th gene carries a somatic mutation in the *i*th tumour sample. For the copy number alteration matrix, the rows still correspond to the tumour samples, but each of our copy number altered genes is represented by two columns, one for amplification and one for deletion. We chose to characterise gene amplification and deletion as two separate variables because they can be thought of as different forms of gene alteration that do not necessarily result in opposing biological effects. We did not further divide the categories into high and low levels of amplifications and losses, as this did not lead to improved results and increased the run time due to the larger data matrix.

We removed all the columns that contain only zeros, since they correspond to genes that carry no form of alteration in any of the tumour samples. We also removed duplicated columns from the copy number alteration matrix. Finally, we filtered out all rows with only zeros as they corresponded to tumour samples without an aberration in any of the genes we tested. These are tumour samples that cannot be distinguished from healthy samples.

As a result, we obtained a 6640×7673 somatic point mutation matrix and a 6640×5477 copy number alteration matrix. The somatic point mutation matrix was used to assess cancer classification using only somatic point-mutated genes. To take into account the copy number alterations, we simply concatenated the two matrices to yield a 6640×13151 binary matrix, in which the first 7673 columns carry information on somatic mutations, while the last 5477 columns carry information on copy number alterations.

### Cancer-type classification and feature selection approaches

We used three different popular machine-learning methods for multiclass classification to classify tumour samples into cancer types. We selected SVMs with a linear kernel [22] and logistic regression [23], both of which are well-known linear methods. We also picked random forests [24] as a third method to test whether its non-linear decision boundaries result in increased discriminatory power between cancer types. Random forests have become popular in bioinformatics in recent years because there is empirical evidence that they perform well in a wide variety of situations [25]. We did not consider deep learning methods, which have recently been proved to be successful in various applications, as they typically require larger sample sizes than what is available to us.

Each algorithm was paired with a feature selection method to identify the top predictor genes. For the SVM, we employed the multiclass version of SVM recursive feature elimination (SVM-RFE) as the feature selection method [26, 27]. SVM-RFE assesses the value of a feature based on its weights in the linear model and discards the least predictive feature in each iteration. The order in which the features are eliminated defines a ranking of the features according to their importance; the least important feature is discarded first while the most important is discarded last. SVM-RFE was implemented with the help of the LiblineaR R package for SVM [28, 29].

To select features in the logistic regression model, we used the glmnet implementation of *L*_1_-regularised logistic regression [30], which follows Zhu and Hastie’s symmetric formulation of multiclass logistic regression [31]. The *L*_1_ regularisation term, which is tuned by a user-defined parameter *λ*, shrinks the coefficients of less relevant features down to zero, thus discarding them.

Random forests measure the importance of each feature by the impact that a random permutation of its values in out-of-the-bag samples has on the classification of those out-of-the-bag samples [23]. To perform feature selection for random forests, we applied an approach from Díaz-Uriarte and de Andrés that utilises these importance measures [32], using the ranger package [33].

The tuning parameters for SVM were optimised via tenfold cross-validation while the out-of-the-bag error of the training data set was used to guide the choice of parameters for the random forest [23].

### Data partitioning and sampling

For an impartial assessment of the classifiers, it is important to test them on a previously unseen data set that is independent of the data set used to train them. If the number of samples is small compared to the number of predictors, many predictors may correlate with the response variable and the results of gene selection can change drastically when different data are used as the training set [34]. To minimise this dependency of gene selection on a single random partitioning of the data into training and test sets, we derived our results from an ensemble of training and test sets.

We first set aside 1/4 of the data (1661 tumour samples) for the final validation of our best classifier. These 1661 tumour samples were randomly picked from the data, with all classes, i.e. tumour types, represented proportionately. We then further divided the remaining 3/4 of the original data set into two sets: a training set and a test data set. We randomly sampled 4/5 of it (again, with all classes represented proportionately) to train the classifiers and tested the optimised classifier on the last 1/5 of the data. This splitting of the data into four parts training and one part testing was repeated 50 times. Any computation we subsequently did was carried out independently on each of these 50 sets. The results were then aggregated by averaging them.

### Assessing classifier performance

We assessed the performance of the classifiers by tracing their overall accuracy as a function of the number of selected predictors. For SVM-RFE and random forests, for each training data set and its corresponding gene ranking by importance, we trained a series of classifiers using an increasing number of the top-ranked genes. We then evaluated the performance of those models using the corresponding test data and averaged the results across the 50 test data sets. For *L*_1_-penalised logistic regression, gene selection was accomplished by varying the regularisation parameter *λ*. We used each of the 50 training data sets to construct a series of logistic regression models by varying *λ*. The corresponding test data sets were then used to estimate the accuracy of each model. For each *λ* value, we averaged the accuracies from the 50 test data sets as well as the number of genes selected.

The overall accuracy of a classifier is not very informative by itself because it does not tell us how well each cancer type is classified. Therefore, we also consider precision and recall. For multiclass classification, precision and recall of a cancer type *i* are defined as:

$$\begin{array}{*{20}l} \text{Precision}_{i} &= \frac{\text{Number of samples correctly classified as cancer type}\ \mathit{i}}{\text{Number of samples classified as cancer type}\ \mathit{i}},  \\ \text{Recall}_{i} &= \frac{\text{Number of samples correctly classified as cancer type}\ \mathit{i}}{\text{Number of samples of cancer type}\ \mathit{i}}.  \end{array} $$

In all calculations, we computed the 95 *%* confidence interval of each quantity by multiplying the standard deviation of its estimate based on the 50 values by $\pm 1.96 / \sqrt {50}$.

### Assessing top feature sets

To verify that the top predictor genes selected by the best classifier are optimal, in the sense that using other genes as the predictor set results in a drop in the performance of the classifier, we compared the performance of the top predictor set selected by our best classifier with that of the second and third best predictor sets, averaged over the 50 training data sets. For each top gene set of size *n*, we took the (*n*+1)th to 2*n*th genes as the second best predictor set, and the (2*n*+1)th to 3*n*th genes as the third best predictor set. We then varied *n* and computed the overall accuracy of the classifier for these three gene sets.

### Stability selection

We analysed the genes selected in the top predictor set as follows. Since we have 50 different training data sets, the list of top genes selected for each of the training sets will, in general, be different. Meinshausen and Bühlmann demonstrated that stability selection, i.e. choosing features that are frequently selected when using different training sets, yields a robust set of predictive features [35]. We followed this approach to find the most frequently selected top genes among the 50 gene lists. Besides examining them in greater detail, we also tested them on the 1661 unseen tumour samples that we set aside at the beginning.

## Results

### Performance of classifiers using somatic point-mutated genes, with and without copy number altered genes

**Fig. 1:**
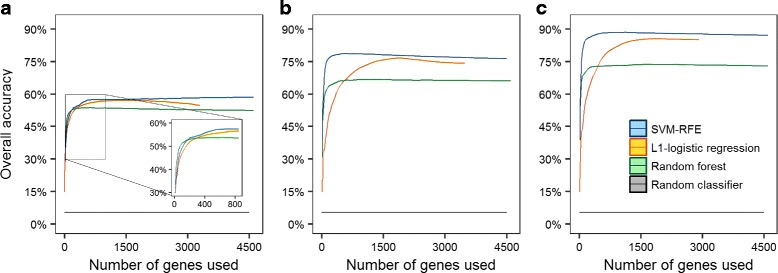
Performance of different classifiers. Using (**a**) only somatic point-mutated genes, (**b**) only copy number altered genes and (**c**) both somatic point-mutated genes and copy number altered genes as the predictors. The mean overall accuracy, with its 95 % confidence interval band, was computed using the results from 50 sets of randomly subsampled training data and their corresponding test data. For SVM-RFE and random forest, we first ranked the genes in decreasing order of their importance, before using an increasing number of them to train and test the classifiers. For *L*_1_-logistic regression, we varied the parameter *λ* to control the number of genes selected. The accuracy of a random classifier is also plotted to provide a baseline for comparison. The random classifier assigns a tumour sample to the different cancer classes with probabilities proportional to the size of those classes in the training data set

Figure 1 summarises the performance of the different classifiers as a function of the number of genes used in the predictor set. We included a random classifier in all the figure panels to provide a baseline for comparison. The random classifier assigns a tumour sample to the different cancer classes with probabilities proportional to the size of those classes in the training data set.

In Fig. 1 a, only somatic point-mutated genes were used as predictors. We observe a sharp increase in the overall accuracy of the classifiers in the initial stage when the number of genes in the predictor set is small. There is, however, a diminishing increase in classifier accuracy with each additional gene used. When the number of genes used reaches 200–300, the overall accuracy of the classifiers starts to level off. When we used only copy number altered genes as the sole predictors of cancer types, we achieved an overall accuracy that is about 20 % better on average than using only somatic point-mutated genes (Fig. 1 b). This observation suggests that combining the two types of gene alterations can further improve performance, which is shown in Fig. 1 c. A comparison of Fig. 1 a, b and c clearly shows that the use of somatic point-mutated genes with copy number altered genes significantly boosts the overall accuracy of the classifiers and gives the best performance.

The linear classifiers generally perform better than the non-linear classifier on the gene alteration data matrices (Fig. 1). In both cases, SVM-RFE and *L*_1_-penalised logistic regression can achieve a higher overall accuracy than random forests. The overall accuracy of SVM-RFE improves more rapidly that that of *L*_1_-penalised logistic regression with the number of genes used. From these observations, we concluded that SVM-RFE is best suited for our data. It achieves the highest overall accuracy of 88.4±0.2 *%* when trained using 900 top-ranked genes consisting of both somatic point-mutated genes and copy number altered genes.

**Fig. 2:**
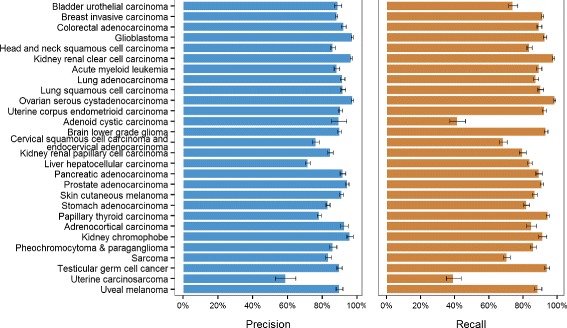
Precision and recall of each of the 28 cancer types for the best SVM model. Here 900 top-ranked genes, consisting of both somatic point mutations and copy number alterations, were used to train the SVM. SVM support vector machine

A closer look at the precision and recall of each cancer type, for the SVM model trained on 900 top-ranked genes, reveals that the classifier does not classify all cancer types equally well (Fig. 2). The precision values are generally high for all the cancer types. With the exception of uterine carcinosarcoma, which has a precision of 59±6 *%*, all other cancer types have precision values exceeding 70 %. The recall values among the different cancer types vary more. Adenoid cystic carcinoma and uterine carcinosarcoma, the two smallest cancer classes, have recall values of 42 ±5 *%* and 40±4 *%*, respectively, while the rest of the cancer types have recall values equal to or greater than 65 %. In other words, a relatively large fraction of tumour samples that have been classified as uterine carcinosarcoma are misclassified, while the proportion of adenoid cystic carcinoma and uterine carcinosarcoma samples that are classified correctly is low. These results suggest that larger sample numbers than the available 55 for adenoid cystic carcinoma and 56 for uterine carcinosarcoma are necessary to improve the classification of these cancer types.

Out of the 28 cancer types, seven of them – glioblastoma, kidney renal clear cell carcinoma, lung squamous cell carcinoma, ovarian serous cystadenocarcinoma, uterine corpus endometrioid carcinoma, prostate adenocarcinoma and kidney chromophobe – have both precision and recall values exceeding 90 %. These results indicate that the somatic mutation and copy number alteration profiles of the top-ranked genes are characteristic of and can correctly differentiate these nine cancers. The precision averaged over the 28 cancer types is 88±2 *%*, while the average recall is 84±2 *%*.

### Performance of SVM-RFE for small predictor gene sets

**Fig. 3:**
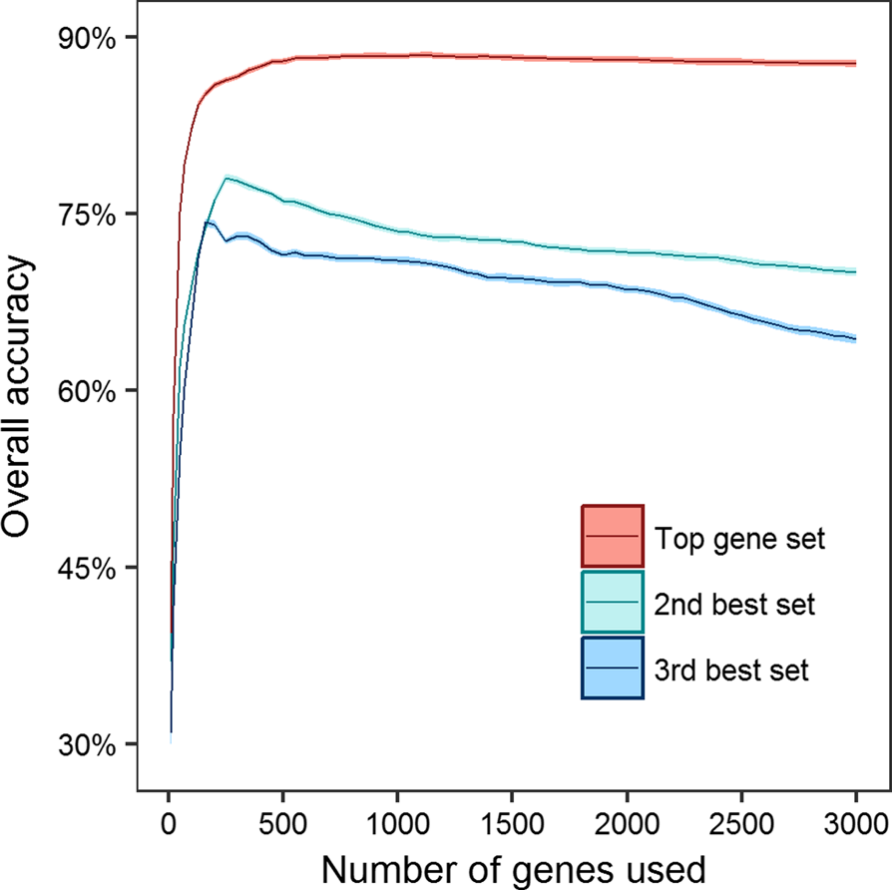
Performance of the top predictor sets when both somatic point-mutated genes and copy number altered genes were used as predictors. The genes were ranked using SVM-RFE. For each top gene set of size *n*, we considered the (*n*+1)th to 2*n*th genes as the second best predictor set, and the (2*n*+1)th to 3*n*th genes as the third best predictor set. We then varied *n* and computed the accuracy of SVM for these three gene sets. SVM support vector machine

When we compared the performance of the top predictor set with that of the second and third best sets, the differences are substantial (Fig. 3). Even when the number of genes used is smaller than 500, the overall accuracy of the top predictor set is still markedly higher than those of the second and third best sets. We computed the overall accuracy of SVM using a top predictor set of size 10, 20, 50, 70 and 100 (Table 2).

**Table 2:** Overall accuracy of SVM for small gene sets selected by RFE

Number of genes used	Only somatic point mutated genes as predictors	Only copy number altered genes as predictors	Somatic point-mutated genes and copy number altered genes
10	28.8±0.5 *%*	39.3±0.8 *%*	40.6±0.9 *%*
20	35.3±0.5 *%*	53.4±0.4 *%*	61.5±0.6 *%*
50	44.3±0.4 *%*	67.7±0.4 *%*	77.7±0.3 *%*
70	47.2±0.4 *%*	71.7±0.3 *%*	81.2±0.3 *%*
100	49.4±0.4 *%*	74.7±0.3 *%*	83.8±0.3 *%*

With both somatic point-mutated genes and copy number altered genes, a mere 50 genes is enough to achieve an overall accuracy of 77.7±0.3 *%*. We carried out stability selection to select 50 genes that are most frequently ranked high in our ranked lists of gene predictors. We found that out of these 50 genes, 18 are associated with somatic point mutations while the remaining 32 are associated with copy number alterations. Furthermore, 13–15 of the 50 genes are cancer driver genes, depending on which list of driver genes we compared to [3, 36, 37]. We note that after manual inspection, we found two non-coding genes in this list, despite our earlier attempt to remove them. Although we were not able to remove all pseudogenes and non-coding genes exhaustively, the remaining ones do not affect the generality of our results presented here. A compilation of the brief descriptions from RefSeqGene [38], as well as the relevant pathways from the Reactome pathway database [39, 40], of our list of 50 genes can be found in Additional file 1.

**Fig. 4:**
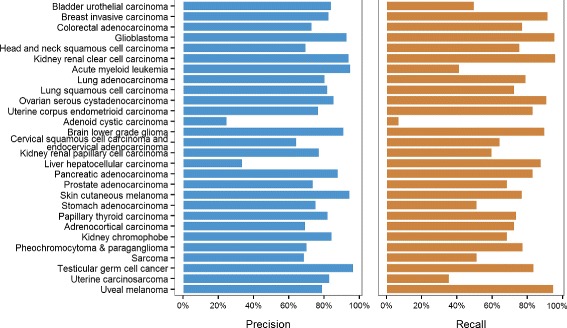
Precision and recall of each of the 28 cancer types, for the SVM model trained with 50 genes chosen via stability selection. The SVM was tested on the 1661 unseen tumour samples that we set aside at the beginning for validation. SVM support vector machine

Training a SVM using these 50 genes and testing it on the 1661 unseen tumour samples that we set aside at the beginning yields an overall accuracy of 77.4 %, which is in good agreement with our results in Table 2. Since the number of genes used here is a small fraction of the 900 genes in the best SVM model, we generally observe a drop in the precision and recall values for each cancer type (Fig. 4). Adenoid cystic carcinoma, which has the smallest sample size in our data set, has 25 % for precision and 7 % for recall. In addition, liver hepatocellular carcinoma has a precision of less than 50 % while bladder urothelial carcinoma, acute myeloid leukaemia and uterine carcinosarcoma have recall values of less than or equal to 50 %. Still, the precision and recall for glioblastoma and kidney renal clear cell carcinoma remain above 90 %. The average precision and recall values fall to 78 % and 72 %, respectively.

### Comparison with other gene panels

A few recent studies have used different methods to select genes for cancer gene panels of different sizes designed to meet slightly different needs. For example, Martinez et al. [41] proposed a panel of 25 somatic point-mutated genes for detecting cancer, but not for identifying the cancer type. These genes were selected based on their relatively high mutation rates in a data set of ten cancer types. Rubio-Perez et al. [42] compiled a list of known cancer driver genes and calculated the cumulative mutational frequency of those genes in a data set of 28 cancer types. They then designed a web interface called OncoPaD that could suggest the most relevant set of genes for different cancer types chosen by the user. These genes were further classified into tiers 1, 2 and 3, with tier 1 genes contributing the most towards the slope of the cumulative mutational frequency distribution.

We compared the effectiveness of our gene selection approach using SVM-RFE and stability selection to the methods used in Martinez et al. and Rubio-Perez et al., in terms of the performance of the gene panels in differentiating tumour types based on gene alteration data. Thus, we used our data set to train two SVM classifiers, one using the 25 genes in Martinez et al. and the other using the 277 genes in the tier 1 set suggested by OncoPaD. We then tested the SVM classifiers on the 1661 unseen tumour samples that we set aside at the beginning. Selecting the same number of somatic point-mutated genes for our gene panel for a fair comparison, our gene panel performed better by about 3–9 % in classifying 28 tumour types, as well as classifying only the tumour types that are common between our and the studies compared (Tables 3 and 4). This result indicates that the gene panels in Martinez et al. and Rubio-Perez et al. are not optimal for differentiating tumour DNA into tumour type. Furthermore, the overall accuracy of our classifier markedly increased when we included copy number alterations, which underscores our earlier results presented in Fig. 1.

**Table 3:** Overall accuracy of the SVM classifier trained using the genes proposed by Martinez et al. and the genes selected via SVM-RFE and stability selection in this study

Classification task	25-gene panel in Martinez et al.	Top 25 SVM-RFE-based SPM genes	Top 25 SVM-RFE-based SPM and CNA genes
28 cancer types of this study	30.4 *%*	39.0 *%*	67.7 *%*
10 cancer types of Martinez et al.	54.6 *%*	57.4 *%*	85.4 *%*

The classifier was tested on 1661 unseen tumour samples

*CNA* copy number altered, *SPM* somatic point-mutated, *SVM* support vector machine, *SVM-RFE* SVM recursive feature eliminatio

**Table 4:** Overall accuracy of the SVM classifier trained using the gene panel proposed by OncoPaD and the genes selected in this study via SVM-RFE and stability selection

Classification task	277 OncoPaD tier 1 genes	Top 277 SVM-RFE-based SPM genes	Top 277 SVM-RFE-based SPM and CNA genes
28 cancer types of this study	49.6 *%*	57.3 *%*	88.1 *%*
19 cancer types common between this study and OncoPaD	56.0 *%*	63.4 *%*	90.3 *%*

The 19 tumour types that are common to our data set and OncoPaD are those labelled 1–11, 13, 14, and 16–21 in Table 1

*CNA* copy number altered, *SPM* somatic point-mutated, *SVM* support vector machine, *SVM-RFE* SVM recursive feature elimination

## Discussion

The goal of this study is to explore the potential of utilising tumour DNA as a cancer diagnostic tool. Thus, we tried to quantify the performance of using genetic alterations in sequenced tumour samples to identify cancer type. This is accomplished by first using machine learning to select informative genes for cancer-type prediction, then using those genes as biomarkers to classify sequenced tumour samples that span 28 different cancer types. In addition, we demonstrated that the selected genes outperform other gene subsets, and that the classification accuracy remains robust when we select genes that are consistently ranked high.

Our results show that by testing 50 genes for somatic point mutations and copy number alterations, the cancer type of an unknown tumour DNA can be identified with an accuracy of around 77 %. Considering the heterogeneity of cancer genomes, it is encouraging that we can achieve such a high accuracy using only a relatively small number of genes. We have seen, however, that the precision and recall for some cancer types can drop to very low values. This is due to two reasons. First, there is an unavoidable trade-off between making the diagnostic tool more useful by increasing the coverage of cancer types on the one hand and reducing the cost of the tool by using fewer genes on the other. Second, cancer types that are consistently classified badly, notably adenoid cystic carcinoma and uterine carcinosarcoma, also have the smallest sample size in our data set. It is expected that with more samples available, predictive diagnostic tools obtained via a similar analysis will become more accurate.

More importantly, our results in Table 2 show that the identification of cancer type using only a small number of somatic point mutations or copy number alterations does not yield satisfactory results. This suggests that a diagnostic tool that depends solely on somatic point mutations might not be very useful. The inclusion of copy number alterations can dramatically improve the results of cancer-type identification. Given the continuing drop in the cost of sequencing, which is the underlying technology for detecting copy number alterations [43], our results suggest that copy number analysis should be part of a cancer diagnostic tool.

Our final 50-gene panel contains genes that were not previously known to be cancer-related. To understand why this may be so, note that the SVM-RFE algorithm selects genes that contribute to differentiating cancer types. These genes may not necessarily be cancer driver genes or genes with high mutation rates. SVM-RFE tends to select complementary genes that, by themselves, may not be the most relevant [26]. Several genes with highly correlated mutation profiles would provide redundant information, even if they are all cancer-related. During the early part of the recursive elimination process, the highly correlated genes will be randomly selected and discarded one by one, until only one of them remains. Hence, the top-ranked genes tend to be less correlated and more complementary. The assortment of genes in our gene panel might explain why they perform better than the genes shortlisted by Martinez et al. and Rubio-Perez et al. Since the gene panels proposed by Martinez et al. and Rubio-Perez et al. are all cancer driver genes, there may be redundancy in the information captured by those genes, hence their suboptimal performance.

On a separate but related note, the panel of 50 genes that we presented should not be regarded as the final word on what a 50-gene panel should be, since in a repeated run of the SVM-RFE algorithm, the correlated genes will be eliminated randomly in a different order, producing a slightly different final gene set. Notwithstanding any difference, the final gene set will still yield a comparable level of performance.

In compiling the genes for our study, we first relied on the MutSig and GISTIC scores, then filtered out pseudogenes and non-coding genes, such that our final list of genes consisted only of protein-coding genes and, therefore, was more interpretable and relatable to other studies. To determine if the removal of the pseudogenes and non-coding genes was justifiable, we ran the whole procedure without removing those genes. Comparing the overall accuracy of SVM on this new data set against our earlier results in Fig. 1, we note that there is a general drop of about 1.4 % in the overall accuracy when pseudogenes and non-coding genes are removed from the somatic point mutation and copy number alteration genes (Additional file 2: Figure A1). On the one hand, this relatively small drop in accuracy shows that we had not filtered out all the useful predictors of cancer types when we removed the pseudogenes and non-coding genes early in our procedure. Since our results were not adversely affected, we think that it is worth removing these genes in exchange for a more interpretable and relatable final gene panel. On the other hand, the drop in accuracy also suggests that at least some of the pseudogenes and non-coding genes might be be helpful in classifying tumour samples into different cancer types. These genes may play a previously overlooked role in cancer progression, such as regulating the expression of cancer-related genes [44].

Since genetic alterations in tumour DNA can be obtained reliably and cost-effectively from circulating tumour DNA (ctDNA), which is released into the bloodstream from viable or ruptured tumour cells, ctDNA is regarded as having potential for cancer-type detection and identification [43, 45]. However, to our knowledge, there is as yet no study that tries to quantify the performance of utilising ctDNA for cancer-type prediction across a broad range of cancer types. Our results in this study can be regarded as providing an estimate of the performance of ctDNA in identifying cancer type under idealised conditions. Such a diagnostic tool would likely not perform as well in reality, because studies have shown that the level of detectable ctDNA is different for different cancer types and in general, the level of ctDNA in the blood correlates with disease stage [46]. This implies that at present, ctDNA might be less useful for certain cancer types and as an early diagnostic tool. We are hopeful that further advances in technology allowing the detection of even lower levels of ctDNA will eventually allow the hypothesised diagnostic tool to approach the level of accuracy we achieved in this study.

Our results and insights should also apply equally well to circulating tumour cells since they contain all the tumour DNA. Hence, the 50 biomarkers that we have identified could potentially be used to determine the cancer types of circulating tumour cells with a comparable level of accuracy.

## Conclusions

Our findings can be interpreted on several levels. First, they increase our understanding of cancer heterogeneity. Genomic alterations in tumours are extremely complex, and large differences occur between tumour genotypes of not only different cancer types but also within the same type. Effectively, judging from somatic alterations, each patient seems to carry a different disease of the genome. However, in clinics, the location of the primary tumour and its histology can be used to give a clear categorisation of patients by cancer type. Thus, out of the numerous mutational and copy number changes that their tumour genomes acquired, some are likely to be involved in disease progression. Machine-learning algorithms, in contrast to manual inspection, offer an efficient means of examining thousands of altered genes across thousands of tumour samples and of dealing with the complexity of genomic alterations and the heterogeneity. In this view, the question of classifying cancer types from genomic alterations can be regarded as deciphering one aspect of the genotype-to-phenotype map.

Second, our study aims at cancer-type prediction using data that are different from those in most of the previous works. Previous studies accumulated evidence that cancer types are distinguishable based on gene expression signatures [4–7] and methylation profiles [8, 9]. To our knowledge, our work is the first to show systematically that cancer types can also be identified from somatic point mutations and copy number alterations. The two earlier studies on genomic alteration data [41, 42] explored related but different questions, and our comparison clearly shows that our top genes outperform their gene sets in distinguishing cancer types.

Finally, from a biological point of view, our results indicate that the most discriminatory power comes from copy number alterations. This indicates that somatic point mutations may have general carcinogenic effects that will be more similar across cancer types. In contrast, copy number variants are more specific for each cancer type.

## Additional files

Additional file 1 Shown are 50 genes selected via stability selection. A list of 50 genes that are most frequently ranked highly by SVM-RFE, their brief descriptions and whether they have been identified as cancer driver genes. (XLSX 24 kb)

Additional file 2 Performance of SVM on data sets with and without pseudogenes and non-coding genes. An additional figure showing the overall accuracy of SVM before and after pseudogenes and non-coding genes were removed from the initial compiled gene lists. (DOCX 28 kb)

## Abbreviations

CNA: Copy number altered
ctDNA: Circulating tumour DNA
SPM: Somatic point-mutated
SVM: Support vector machine
SVM-RFE: Support vector machine with recursive feature selection
TCGA: The Cancer Genome Atlas
