# Supplementary material for: Predicting cancer type from tumour DNA signatures
Source: Genome Med. 2017 Nov 28;9:104. doi: 10.1186/s13073-017-0493-2 (PMC5706302; doi:10.1186/s13073-017-0493-2)
Supplement: Supplementary file 2 — Performance of SVM on data sets with and without pseudogenes and non-coding genes. An additional figure showing the overall accuracy of SVM before and after pseudogenes and non-coding genes were removed from the initial compiled gene lists. (DOCX 28 kb) [file 13073_2017_493_MOESM2_ESM.docx]

**Additional File 4**


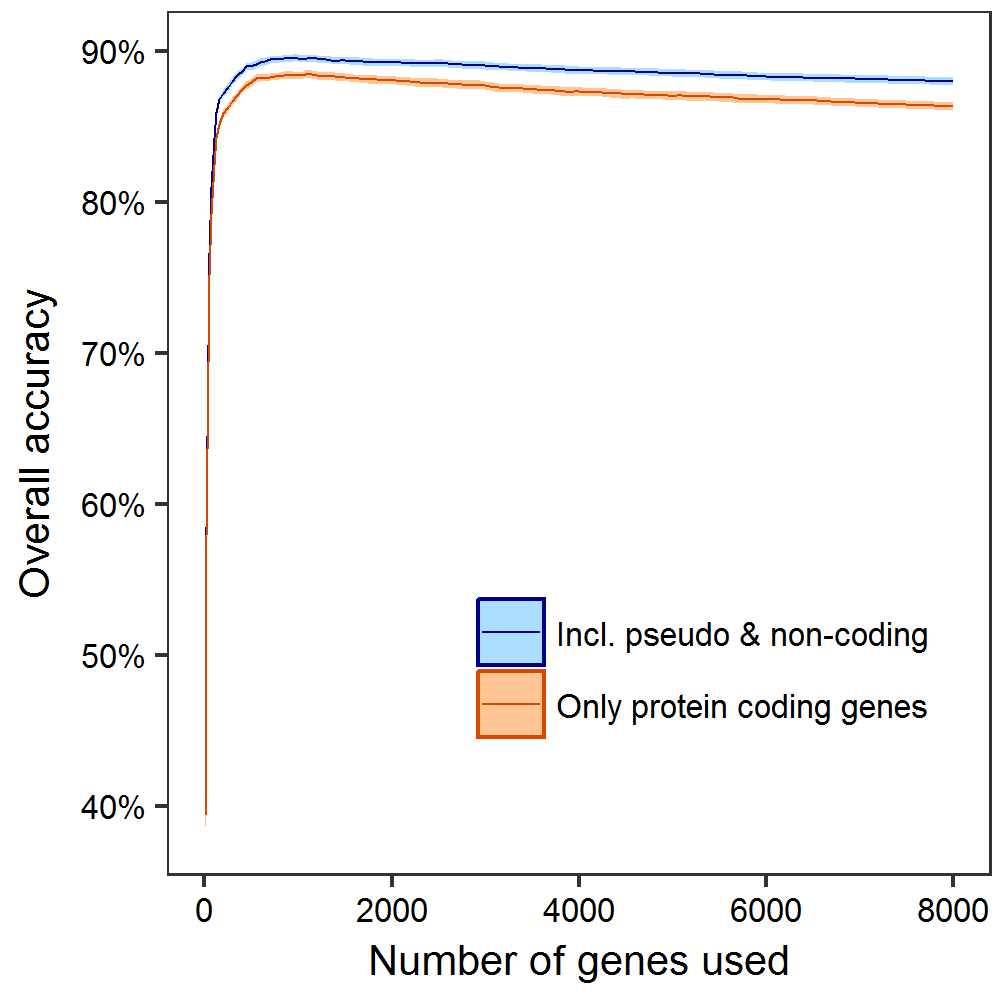


**Figure A1: Performances of SVM on datasets with and without pseudogenes and non-coding genes**, when both somatic point mutated genes and copy number altered genes were used as predictors. There is a general drop of around 1.4% in the overall accuracy after 1074 pseudogenes and non-coding genes were removed from the original gene list. All the genes were first compiled based on the MutSig and GISTIC scores.
